# *O*,*O*′-Diethyl {(*Z*)-[(2-chloro­phen­yl)(cyano)methyl­ene]amino­oxy}thio­phospho­nate

**DOI:** 10.1107/S160053680900693X

**Published:** 2009-03-06

**Authors:** Qiong Gao, Qing-xia Zhao, Pu-hai Wang

**Affiliations:** a College of Life Science and Pharmaceutical Engineering, Nanjing University of Technology, No. 5 Xinmofan Road, Nanjing 210009, People’s Republic of China; b Department of Medicinal Chemistry, Jiangsu Provincial Institute of Materia Medica, Nanjing University of Technology, No. 26 Majia Street, Nanjing 210009, People’s Republic of China

## Abstract

The title mol­ecule, C_12_H_14_ClN_2_O_3_PS, has a *cis* configuration with respect to the C=N bond. Inter­molecular C—H⋯O inter­actions inter­connect the mol­ecules into chains along the *c* axis. The chains are further connected into a two-dimensional network parallel to the *bc* plane by weak π–π inter­actions between adjacent aromatic rings (centroid–centroid distance = 3.772Å).

## Related literature

For the insectidal activity of the title compound, see: Hudson & Obudho (1972 ▶); Le Berre *et al.* (1972 ▶). For its preparation and reactivity, see: Walter & Clifton (1973 ▶); Wang *et al.* (1996 ▶).
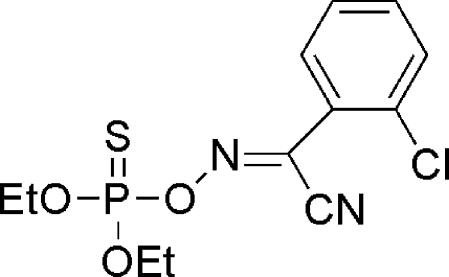


## Experimental

### 

#### Crystal data

C_12_H_14_ClN_2_O_3_PS
*M* *_r_* = 332.73
Monoclinic, 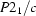

*a* = 10.518 (2) Å
*b* = 20.215 (4) Å
*c* = 7.9650 (16) Å
β = 110.11 (3)°
*V* = 1590.3 (6) Å^3^
*Z* = 4
Mo *K*α radiation
μ = 0.48 mm^−1^
*T* = 293 K
0.30 × 0.20 × 0.10 mm

#### Data collection

Enraf–Nonius CAD-4 diffractometer
Absorption correction: ψ scan (North *et al.*, 1968 ▶) *T*_min_ = 0.870, *T*_max_ = 0.954
2889 measured reflections
2889 independent reflections
1981 reflections with *I* > 2σ(*I*)
3 standard reflections frequency: 120 min intensity decay: 1.0%

#### Refinement

*R*[*F* ^2^ > 2σ(*F* ^2^)] = 0.061
*wR*(*F* ^2^) = 0.192
*S* = 1.09
2889 reflections
183 parameters
H-atom parameters constrained
Δρ_max_ = 0.31 e Å^−3^
Δρ_min_ = −0.39 e Å^−3^

### 

Data collection: *CAD-4 EXPRESS* (Enraf–Nonius, 1994 ▶); cell refinement: *CAD-4 EXPRESS*; data reduction: *XCAD4* (Harms & Wocadlo, 1995 ▶); program(s) used to solve structure: *SHELXS97* (Sheldrick, 2008 ▶); program(s) used to refine structure: *SHELXL97* (Sheldrick, 2008 ▶); molecular graphics: *SHELXTL* (Sheldrick, 2008 ▶); software used to prepare material for publication: *SHELXL97*.

## Supplementary Material

Crystal structure: contains datablocks global, I. DOI: 10.1107/S160053680900693X/fb2135sup1.cif

Structure factors: contains datablocks I. DOI: 10.1107/S160053680900693X/fb2135Isup2.hkl

Additional supplementary materials:  crystallographic information; 3D view; checkCIF report

## Figures and Tables

**Table 1:** Hydrogen-bond geometry (Å, °)

*D*—H⋯*A*	*D*—H	H⋯*A*	*D*⋯*A*	*D*—H⋯*A*
C2—H2*A*⋯O1^i^	0.97	2.58	3.396 (6)	142

Symmetry code: (i) 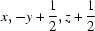.

**Fig. 1.**
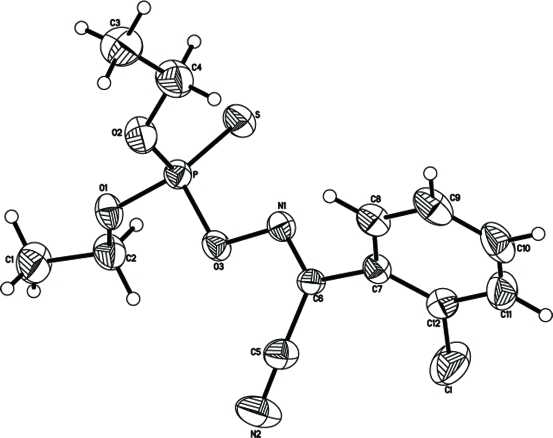
The molecular structure of the title molecule with the atom-numbering scheme. The displacement ellipsoids are drawn at the 30% probability level.

**Fig. 2.**
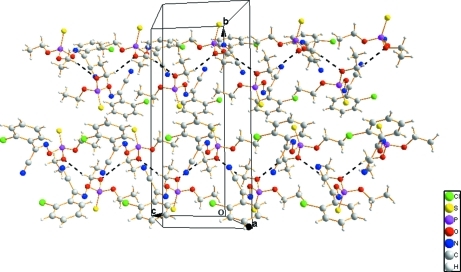
A packing diagram of the title structure. The dashed lines represent weak C—H···O interactions.

## supplementary crystallographic information

### Comment

*O*,*O*-diethyl-*O*-(2-chlorophenylglyoxylonitrile oximino)thiophosphate (Chlorphoxim) was shown to be efficient against adult mosquitoes as well as agricultural insects (Hudson *et al.*, 1972). It was also successfully tested against the larvae of the blackfly (Simulium damnosum), the insect vector of human onchocerciasis in West Africa (Le Berre *et al.*, 1972). The title substance combined with niclosamide exhibited significant molluscicidal synergism against snails (Oncomelania hupensis) (Wang *et al.*, 1996). The synthesis of the title compound has been described by Walter *et al.* (1973). As a part of our own studies in this area, we report here its crystal structure.

The title molecule shows a *cis* configuration (Fig. 1) on the C═N bond. The molecules are linked into chains along the axis c *via* weak intermolecular C—H···O interactions (Fig. 2, Tab. 1). The chains are further connected into a two-dimensional network *via* weak π-π electron interactions between the adjacent phenyl rings: The centroid-centroid distance is 3.772 Å.

### Experimental

Sodium, 2.30 g (0.1 mol), reacted with 50 ml of absolute ethanol in order to get sodium ethoxide solution. 15.20 g (0.1 mol) of 2-chlorophenylacetonitrile, was added dropwise to the cooled sodium ethoxide (about 278 K) and then 10.30 g (0.1 mol) of butyl nitrite was added dropwise. The mixture was stirred at room temperature for 1 h under reduced pressure until the volume was reduced to 30 ml. Then 50 ml of ethyl ether was added, and the precipitated solid was filtered off and washed with ethyl ether to afford sodium 2-chlorophenylglyoxylonitrile oxime (11.3 g, 56%). Diethyl phosphorochloridothionate, 5.70 g (0.03 mol), was added dropwise to 6.00 g (0.03 mol) of sodium 2-chlorophenylglyoxylonitrile oxime, which was suspended in 20 ml of dry acetone. The mixture was stirred for 1 h. Thin layered chromatography using petroleum ether and ethyl acetate as expanding solvent indicated just one point. The mixture was then concentrated under reduced pressure and 50 ml of water was added to the residue. The precipitated solid that had appeared was filtered off, washed thoroughly with absolute ethanol, dried and recrystallized from petroleum ether to afford the title compound (8.9 g, yield 89%) as a white crystalline solid. The title crystals suitable for X-ray diffraction were obtained by slow evaporation of the acetone solution. The average size of the block-like crystals is 0.2 × 0.2 × 0.2 mm.

### Refinement

The aryl and methylene H atoms were situated into idealized positions though the aryl H atoms were clearly discernible in the difference electron density map. After the refinement with these H atoms whose parameters were fully constrained had converged the electron density map revealed that the methyl H atoms were not disordered. They were also situated into the idealized positions and constrained. *C*—H_methyl_, *C*—H_methylene_, *C*—H_aryl_ = 0.96, 0.97, 0.93 Å, *U*_iso_H_methyl_=1.5*U*_eq_C_methyl_, *U*_iso_H_methylene_=1.2*U*_eq_C_methylene_, *U*_iso_H_aryl_=1.2*U*_eq_C_aryl_.

### Crystal data

**Table:**

C_12_H_14_ClN_2_O_3_PS	*F*(000) = 688
*M**_r_* = 332.73	*D*_x_ = 1.390 Mg m^−^^3^
Monoclinic, *P*2_1_/*c*	Melting point: 341.5 K
Hall symbol: -P 2ybc	Mo *K*α radiation, λ = 0.71073 Å
*a* = 10.518 (2) Å	Cell parameters from 25 reflections
*b* = 20.215 (4) Å	θ = 9.0–13.0°
*c* = 7.9650 (16) Å	µ = 0.48 mm^−^^1^
β = 110.11 (3)°	*T* = 293 K
*V* = 1590.3 (6) Å^3^	Block, colourless
*Z* = 4	0.30 × 0.20 × 0.10 mm

### Data collection

**Table:**

Enraf–Nonius CAD-4 diffractometer	1981 reflections with *I* > 2σ(*I*)
Radiation source: fine-focus sealed tube	*R*_int_ = 0.0000
graphite	θ_max_ = 25.3°, θ_min_ = 2.0°
ω/2θ scans	*h* = −12→11
Absorption correction: ψ scan (North *et al.*, 1968)	*k* = 0→24
*T*_min_ = 0.870, *T*_max_ = 0.954	*l* = 0→9
2889 measured reflections	3 standard reflections every 120 min
2889 independent reflections	intensity decay: 1.0%

### Refinement

**Table:**

Refinement on *F*^2^	Primary atom site location: structure-invariant direct methods
Least-squares matrix: full	Secondary atom site location: difference Fourier map
*R*[*F*^2^ > 2σ(*F*^2^)] = 0.061	Hydrogen site location: difference Fourier map
*wR*(*F*^2^) = 0.192	H-atom parameters constrained
*S* = 1.09	*w* = 1/[σ^2^(*F*_o_^2^) + (0.0957*P*)^2^ + 1.1623*P*] where *P* = (*F*_o_^2^ + 2*F*_c_^2^)/3
2889 reflections	(Δ/σ)_max_ < 0.001
183 parameters	Δρ_max_ = 0.31 e Å^−^^3^
0 restraints	Δρ_min_ = −0.38 e Å^−^^3^

### Special details

**Table:**

Geometry. All e.s.d.'s (except the e.s.d. in the dihedral angle between two l.s. planes) are estimated using the full covariance matrix. The cell e.s.d.'s are taken into account individually in the estimation of e.s.d.'s in distances, angles and torsion angles; correlations between e.s.d.'s in cell parameters are only used when they are defined by crystal symmetry. An approximate (isotropic) treatment of cell e.s.d.'s is used for estimating e.s.d.'s involving l.s. planes.
Refinement. Refinement of *F*^2^ against ALL reflections. The weighted *R*-factor *wR* and goodness of fit *S* are based on *F*^2^, conventional *R*-factors *R* are based on *F*, with *F* set to zero for negative *F*^2^. The threshold expression of *F*^2^ > σ(*F*^2^) is used only for calculating *R*-factors(gt) *etc*. and is not relevant to the choice of reflections for refinement. *R*-factors based on *F*^2^ are statistically about twice as large as those based on *F*, and *R*- factors based on ALL data will be even larger.

### Fractional atomic coordinates and isotropic or equivalent isotropic displacement parameters (Å^2^)

**Table:**

	*x*	*y*	*z*	*U*_iso_*/*U*_eq_	
Cl	0.9833 (2)	0.10567 (10)	1.4318 (2)	0.1103 (7)	
S	0.44401 (12)	0.05415 (6)	0.77933 (17)	0.0620 (4)	
P	0.49482 (11)	0.14026 (6)	0.73194 (14)	0.0468 (3)	
O1	0.3963 (3)	0.19852 (15)	0.7185 (4)	0.0562 (8)	
O2	0.5248 (4)	0.15378 (16)	0.5556 (4)	0.0687 (9)	
O3	0.6273 (3)	0.16956 (14)	0.8892 (4)	0.0518 (7)	
N1	0.7388 (3)	0.12634 (16)	0.9243 (4)	0.0477 (8)	
N2	0.8474 (5)	0.2615 (3)	1.2012 (8)	0.0939 (16)	
C1	0.2127 (6)	0.2550 (3)	0.7678 (8)	0.0898 (18)	
H1B	0.1664	0.2624	0.8511	0.135*	
H1C	0.1501	0.2376	0.6583	0.135*	
H1D	0.2493	0.2960	0.7441	0.135*	
C2	0.3231 (5)	0.2074 (3)	0.8440 (7)	0.0690 (13)	
H2A	0.3843	0.2236	0.9575	0.083*	
H2B	0.2864	0.1653	0.8645	0.083*	
C3	0.5814 (7)	0.1315 (3)	0.2996 (7)	0.0884 (18)	
H3A	0.6215	0.0996	0.2437	0.133*	
H3B	0.6290	0.1727	0.3118	0.133*	
H3C	0.4881	0.1379	0.2273	0.133*	
C4	0.5897 (7)	0.1079 (3)	0.4736 (8)	0.0847 (17)	
H4A	0.5460	0.0650	0.4623	0.102*	
H4B	0.6839	0.1027	0.5482	0.102*	
C5	0.8423 (4)	0.2119 (2)	1.1322 (6)	0.0599 (12)	
C6	0.8431 (4)	0.14942 (19)	1.0451 (5)	0.0436 (9)	
C7	0.9702 (4)	0.1113 (2)	1.0871 (6)	0.0475 (9)	
C8	1.0192 (5)	0.0965 (2)	0.9514 (7)	0.0590 (11)	
H8A	0.9715	0.1105	0.8356	0.071*	
C9	1.1372 (5)	0.0612 (3)	0.9845 (9)	0.0759 (15)	
H9A	1.1686	0.0512	0.8917	0.091*	
C10	1.2084 (5)	0.0411 (3)	1.1563 (10)	0.0865 (19)	
H10A	1.2885	0.0174	1.1797	0.104*	
C11	1.1627 (6)	0.0554 (3)	1.2906 (9)	0.0836 (17)	
H11A	1.2118	0.0420	1.4065	0.100*	
C12	1.0435 (5)	0.0899 (2)	1.2575 (6)	0.0642 (12)	

### Atomic displacement parameters (Å^2^)

**Table:**

	*U*^11^	*U*^22^	*U*^33^	*U*^12^	*U*^13^	*U*^23^
Cl	0.1224 (14)	0.1444 (17)	0.0590 (8)	0.0170 (12)	0.0247 (9)	0.0086 (9)
S	0.0607 (7)	0.0481 (6)	0.0785 (8)	−0.0014 (5)	0.0256 (6)	−0.0008 (6)
P	0.0463 (6)	0.0495 (6)	0.0467 (6)	0.0072 (5)	0.0187 (5)	0.0010 (5)
O1	0.0543 (16)	0.0572 (18)	0.0650 (18)	0.0168 (14)	0.0309 (14)	0.0129 (14)
O2	0.084 (2)	0.070 (2)	0.063 (2)	0.0186 (18)	0.0395 (18)	0.0105 (16)
O3	0.0433 (14)	0.0463 (16)	0.0648 (18)	0.0068 (12)	0.0173 (13)	−0.0028 (14)
N1	0.0452 (18)	0.0482 (19)	0.0533 (19)	0.0053 (15)	0.0216 (16)	0.0008 (15)
N2	0.077 (3)	0.080 (3)	0.124 (4)	−0.006 (3)	0.035 (3)	−0.046 (3)
C1	0.081 (4)	0.111 (5)	0.081 (4)	0.041 (4)	0.033 (3)	0.001 (3)
C2	0.079 (3)	0.072 (3)	0.065 (3)	0.020 (3)	0.036 (3)	0.005 (2)
C3	0.120 (5)	0.096 (4)	0.070 (3)	−0.002 (4)	0.060 (4)	0.004 (3)
C4	0.106 (4)	0.081 (4)	0.078 (4)	0.016 (3)	0.045 (3)	−0.001 (3)
C5	0.044 (2)	0.068 (3)	0.067 (3)	−0.004 (2)	0.018 (2)	−0.014 (2)
C6	0.048 (2)	0.041 (2)	0.042 (2)	−0.0015 (16)	0.0155 (17)	−0.0039 (16)
C7	0.041 (2)	0.043 (2)	0.058 (2)	−0.0001 (17)	0.0169 (18)	0.0001 (19)
C8	0.056 (3)	0.052 (3)	0.075 (3)	−0.002 (2)	0.031 (2)	−0.004 (2)
C9	0.063 (3)	0.059 (3)	0.121 (5)	0.001 (2)	0.051 (3)	−0.010 (3)
C10	0.053 (3)	0.055 (3)	0.142 (6)	0.008 (2)	0.020 (3)	−0.001 (3)
C11	0.065 (3)	0.070 (3)	0.093 (4)	0.005 (3)	−0.002 (3)	0.009 (3)
C12	0.062 (3)	0.060 (3)	0.062 (3)	0.001 (2)	0.010 (2)	−0.006 (2)

### Geometric parameters (Å, °)

**Table:**

Cl—C12	1.743 (5)	C3—H3A	0.9600
S—P	1.8966 (16)	C3—H3B	0.9600
P—O1	1.548 (3)	C3—H3C	0.9600
P—O2	1.566 (3)	C4—H4A	0.9700
P—O3	1.631 (3)	C4—H4B	0.9700
O1—C2	1.467 (5)	C5—C6	1.442 (6)
O2—C4	1.435 (6)	C6—C7	1.478 (5)
O3—N1	1.412 (4)	C7—C12	1.380 (6)
N1—C6	1.273 (5)	C7—C8	1.381 (6)
N2—C5	1.136 (6)	C8—C9	1.376 (7)
C1—C2	1.469 (7)	C8—H8A	0.9300
C1—H1B	0.9600	C9—C10	1.377 (9)
C1—H1C	0.9600	C9—H9A	0.9300
C1—H1D	0.9600	C10—C11	1.347 (9)
C2—H2A	0.9700	C10—H10A	0.9300
C2—H2B	0.9700	C11—C12	1.378 (7)
C3—C4	1.440 (7)	C11—H11A	0.9300
			
O1—P—O2	98.17 (17)	O2—C4—C3	110.1 (5)
O1—P—O3	98.71 (16)	O2—C4—H4A	109.6
O2—P—O3	104.07 (18)	C3—C4—H4A	109.6
O1—P—S	118.96 (13)	O2—C4—H4B	109.6
O2—P—S	119.75 (15)	C3—C4—H4B	109.6
O3—P—S	113.90 (12)	H4A—C4—H4B	108.2
C2—O1—P	122.8 (3)	N2—C5—C6	177.0 (5)
C4—O2—P	124.7 (3)	N1—C6—C5	122.6 (4)
N1—O3—P	111.1 (2)	N1—C6—C7	117.2 (3)
C6—N1—O3	111.4 (3)	C5—C6—C7	120.1 (4)
C2—C1—H1B	109.5	C12—C7—C8	118.0 (4)
C2—C1—H1C	109.5	C12—C7—C6	122.8 (4)
H1B—C1—H1C	109.5	C8—C7—C6	119.2 (4)
C2—C1—H1D	109.5	C9—C8—C7	121.1 (5)
H1B—C1—H1D	109.5	C9—C8—H8A	119.4
H1C—C1—H1D	109.5	C7—C8—H8A	119.4
O1—C2—C1	108.9 (4)	C8—C9—C10	119.4 (5)
O1—C2—H2A	109.9	C8—C9—H9A	120.3
C1—C2—H2A	109.9	C10—C9—H9A	120.3
O1—C2—H2B	109.9	C11—C10—C9	120.3 (5)
C1—C2—H2B	109.9	C11—C10—H10A	119.8
H2A—C2—H2B	108.3	C9—C10—H10A	119.8
C4—C3—H3A	109.5	C10—C11—C12	120.4 (5)
C4—C3—H3B	109.5	C10—C11—H11A	119.8
H3A—C3—H3B	109.5	C12—C11—H11A	119.8
C4—C3—H3C	109.5	C11—C12—C7	120.7 (5)
H3A—C3—H3C	109.5	C11—C12—Cl	119.7 (4)
H3B—C3—H3C	109.5	C7—C12—Cl	119.6 (4)
			
O2—P—O1—C2	174.0 (4)	C5—C6—C7—C12	58.7 (6)
O3—P—O1—C2	−80.3 (4)	N1—C6—C7—C8	55.6 (5)
S—P—O1—C2	43.2 (4)	C5—C6—C7—C8	−120.9 (5)
O1—P—O2—C4	−166.5 (4)	C12—C7—C8—C9	0.2 (7)
O3—P—O2—C4	92.3 (5)	C6—C7—C8—C9	179.9 (4)
S—P—O2—C4	−36.3 (5)	C7—C8—C9—C10	−0.7 (7)
O1—P—O3—N1	−177.2 (2)	C8—C9—C10—C11	0.2 (8)
O2—P—O3—N1	−76.5 (3)	C9—C10—C11—C12	0.6 (9)
S—P—O3—N1	55.7 (3)	C10—C11—C12—C7	−1.1 (8)
P—O3—N1—C6	179.0 (3)	C10—C11—C12—Cl	177.9 (5)
P—O1—C2—C1	−164.6 (4)	C8—C7—C12—C11	0.7 (7)
P—O2—C4—C3	170.6 (4)	C6—C7—C12—C11	−179.0 (4)
O3—N1—C6—C5	0.4 (5)	C8—C7—C12—Cl	−178.3 (3)
O3—N1—C6—C7	−176.0 (3)	C6—C7—C12—Cl	2.0 (6)
N1—C6—C7—C12	−124.7 (5)		

### Hydrogen-bond geometry (Å, °)

**Table:**

*D*—H···*A*	*D*—H	H···*A*	*D*···*A*	*D*—H···*A*
C2—H2A···O1^i^	0.97	2.58	3.396 (6)	142

Symmetry codes: (i) *x*, −*y*+1/2, *z*+1/2.
